# Immunity and Fibrogenesis: The Role of Th17/IL-17 Axis in HBV and HCV-induced Chronic Hepatitis and Progression to Cirrhosis

**DOI:** 10.3389/fimmu.2017.01195

**Published:** 2017-09-28

**Authors:** Feliciano Chanana Paquissi

**Affiliations:** ^1^Department of Medicine, Clínica Girassol, Luanda, Angola

**Keywords:** Th17 Cells, interleukin-17, TGF-β, fibrogenesis, cirrhosis, chronic viral hepatitis, hepatitis C virus, hepatitis B virus

## Abstract

Cirrhosis is a common final pathway for most chronic liver diseases; representing an increasing burden worldwide and is associated with increased morbidity and mortality. Current evidence has shown that, after an initial injury, the immune response has a significant participation in the ongoing damage, and progression from chronic viral hepatitis (CVH) to cirrhosis, driving the activation and maintenance of main fibrogenic pathways. Among immune deregulations, those related to the subtype 17 of T helper lymphocytes (Th17)/interleukin-17 (IL-17) axis have been recognized as key immunopathological and prognostic elements in patients with CVH. The Th17/IL-17 axis has been found involved in several points of fibrogenesis chain from the activation of stellate cells, increased expression of profibrotic factors as TGF-β, promotion of the myofibroblastic or epithelial–mesenchymal transition, stimulation of the synthesis of collagen, and induction of imbalance between matrix metalloproteinases and tissue inhibitors of metalloproteinases (TIMPs). It also promotes the recruitment of inflammatory cells and increases the expression of proinflammatory cytokines such as IL-6 and IL-23. So, the Th17/IL-17 axis is simultaneously the fuel and the flame of a sustained proinflammatory and profibrotic environment. This work aims to present the immunopathologic and prognostic role of the Th17/IL-17 axis and related pathways in fibrogenesis and progression to cirrhosis in patients with liver disease due to hepatitis B virus (HBV) and hepatitis C virus (HCV).

## Introduction

Liver cirrhosis is a common final pathway for most chronic liver diseases; and is increasingly becoming a major cause of global health burden, being responsive for high morbidity and mortality worldwide. Chronic viral hepatitises (CVHs) are the leading cause of cirrhosis, also with increasing burden worldwide (1). According to the report of Global Burden of Disease Study 2013, between 1990 and 2013, occurred a 63% increase in the global viral hepatitis deaths, passing from the tenth (in 1990) to seventh (in 2013) leading cause of death worldwide (1). There was also an increase in attributable years of life lost, years living with a disability (34% for each), and in the absolute burden of the disease (1). In parallel, despite significant progress in the treatment of CVH during the last decades, the number of deaths from cirrhosis and hepatocellular carcinoma (HCC) increased in the last 20 years (2, 3).

**Figure 1:**
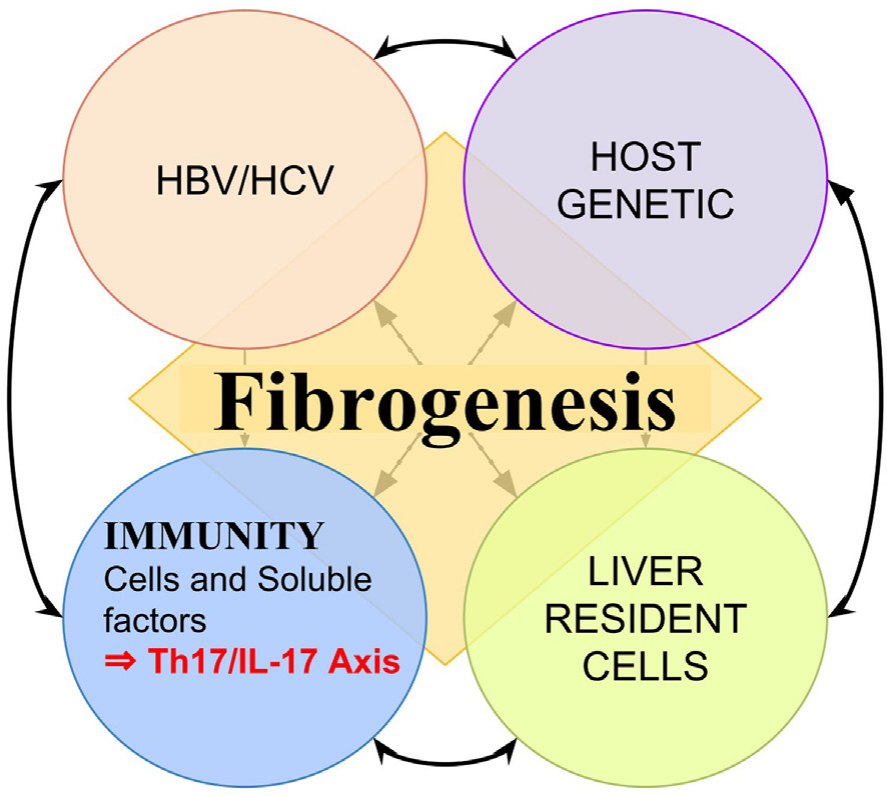
Representation of multiple factors influencing liver fibrogenesis. Fibrogenesis is a result of continuous interaction between pathogen-related factors such as hepatitis B virus (HBV) protein X and hepatitis C virus (HCV) core antigen (4–6, 27), the host genetic such as certain HLA haplotypes (7, 8), liver resident cells, and the immune system such as certain interleukin polymorphisms (9–13, 15).

Currently, it is known that after the initial injury, before cirrhosis is established, multiple pathways of fibrogenesis are activated as a result of continuous interaction between pathogen-related factors (4–6), the host genetic such as certain HLA haplotypes and cytokines gene polymorphisms (7–11), liver resident cells, and the immune system (9–13) (Figure 1). Indeed, cirrhosis is a reflection of sustained injuries and constant and exaggerated attempts of tissue repair, in which the immune system has crucial participation (14–16). The inappropriate immune response have an influence on the activation and maintenance of fibrogenic pathways and progression from CVH to cirrhosis (17–20). Multiple imbalances in immune response, either in cellular or soluble factors, have been associated with the evolution to cirrhosis in viral hepatitis (21, 22). In addition, the immune response influences on viral persistence and response to treatment (11, 23). Thus, efforts have been made in research and clinical practice, aiming to get a better comprehension of the immunological mechanisms underlying these pathways, their exploration as immune biomarkers to predict outcomes, response to treatment, as well as explore their potential as targets for adjuvant therapeutics (24–26).

Among the elements of the immune response, the subtype 17 of T helper lymphocytes (Th17) has gained space as a biomarker with noteworthy performance in the prediction of progression to cirrhosis in liver diseases, particularly in CVH (20, 28). Therefore, the cytokines secreted by Th17, particularly the interleukin-17 (IL-17), also have been implicated in the activation of fibrogenic pathways and progression to cirrhosis (13, 17). Th17 cells have a predominantly effector functional profile, being responsible for the immune surveillance, but are also involved in the pathogenesis of many autoimmune diseases and in the mechanisms of fibrosis in many organs, after the initial injury (29–31).

In CVH, the Th17/IL-17 axis expresses a sustained aggression, and especially a proinflammatory and profibrotic environment with recruitment and activation of other cells, promoting this way, more tissue injury and dysfunctional reparative responses (13, 17, 32, 33). This review aims to summarize the existing knowledge on the immunopathologic and prognostic role of the Th17/IL-17 axis and related pathways in fibrogenesis and progression to cirrhosis in patients with liver disease due to hepatitis B virus (HBV) and hepatitis C virus (HCV).

## An Overview of General Inflammatory Biomarkers as Outcome Predictors in CVH

General Inflammatory biomarkers, such as C-reactive protein (CRP), and interleukin-6, have been associated with poor outcomes on viral hepatitis (11, 26, 34). High levels of interleukin-6 are related to adverse outcomes in the CVH, including greater severity (35, 36), worse response to treatment and viral persistence (11, 26), and evolution to cirrhosis, HCC, or death (35, 36). In a study including 149 patients with CHC (and 17 controls) who underwent 12 weeks’ treatment with pegylated IFN-α2b and ribavirin, serum IL-6 levels were significantly higher in CHC patients than in controls, and a high pretreatment IL-6 was associated with lower rates of sustained virologic response (SVR) (52 vs. 79%; *P* = 0.012) (26). In addition, SVR was accompanied by a significant decrease in IL-6 levels (from 2.7 to 1.5 pg/ml; *P* = 0.029) at 4 weeks of treatment, remaining significantly lower in responder than in non-responder (26). In other studies, it was observed that certain polymorphisms in IL-6 or its promoter are associated with lower rate of spontaneous clearance (11), and increased risk of HCC in patients with CVH-related cirrhosis (10, 37). Many other proinflammatory cytokines, including interleukin IL-1β, IL-18, and TNF-α have been associated with increased risk of progression to cirrhosis (38–40). Certain polymorphisms in TNF-α are also associated with higher risk of becoming chronic carriers of viral infection, progression to cirrhosis and HCC (15, 40).

In addition to the soluble factors, cells of the inflammatory response as neutrophils (19, 41) and monocytes have been found associated with worse outcomes in viral hepatitis. All these elements (cells and soluble factors) are part of a context in which the Th17/IL-17 axis imbalances work as crucial elements to feed and keep this proinflammatory and profibrotic microenvironment (20, 22, 42–44), as described in the next sections.

## The Th17/IL-17 Axis and Outcome Prediction in CVH

The inappropriate immune response at the level of the Th17/IL-17 axis exerts influence in maintaining the fibrogenic pathways and progression from CVH to cirrhosis (9, 44). The fibrogenic role of IL-17, the main cytokine of Th17, has been appointed in increasing number of publications in recent years (13, 45, 46). In addition to its direct effect on fibrogenesis, the Th17/IL-17 axis is the fuel to sustain the proinflammatory environment, with the recruitment of the other cells and stimulation of the synthesis of other soluble factors (29, 33, 47–50). At the same time, Th17/IL-17 axis expresses itself, a response to a sustained proinflammatory stimulus (51, 52). So, we can understand the broad scope of the elements of this axis on the CVH, predicting outcomes in different points of the disease, from spontaneous healing (53), response to treatment (23, 54, 55), occurrence of acute decompensations (56), progression to cirrhosis (9, 28), and HCC (53), including post-transplant recurrence (57), as detailed in this section.

### The Th17/IL-17 Axis and Severity of CVH

The Th17/IL-17 axis is associated with high disease severity in CVHs, as found in a study where patients with chronic hepatitis B (CHB) had higher percentage of Th17 cells in peripheral blood than healthy controls (HCs) (1.53 vs. 0.92%); and among patients there was a positive correlation between peripheral Th17 cells and serum alanine aminotransferase (ALT) (58). In another study, including 96 patients with HBV-related conditions, the serum IL-17 concentration, intrahepatic, and peripheral Th17 cells were significantly higher in both CHB and acute-on-chronic liver failure (ACLF) patients than in asymptomatic surface antigen carriers (AsC) or HCs (59). Th17 cells increased progressively as aggravated the immune inflammation from AsC, CHB, to ACLF, with a positive correlation with severity markers (INR and MELD score) (59). Compared with HCs, patients with CHC had also higher proportions of Th17 cells either circulating (1.56 vs. 0.96%) or infiltrating the liver (16.08 vs. 0.82/hpf), associated with higher serum IL-17 levels (84.86 vs. 60.52 pg/mL) (42). In these patients, both peripheral and intrahepatic Th17 cells correlated with the severity of liver inflammation and damage (28, 42).

### The Th17/IL-17 Axis and Progression to Cirrhosis in CVH

Concerning the progression to cirrhosis, in a study including 173 patients with chronic liver diseases, there was a significant increase in serum IL-17 protein and IL -17 mRNA levels in chronic HBV-related conditions than in HCs (*P* < 0.001); and patients with cirrhosis exhibited the highest serum IL-17 and IL-17 mRNA in peripheral blood mononuclear cells (PBMCs) (44). In another study with 101 patients with HBV-related LC or CHB; peripheral Th17 cells increased significantly in patients with cirrhosis as increased disease severity (mean: 3.51, 3.94, and 4.46; for Child–Pugh A, B, and C, respectively) (28). In intrahepatic tissue, a study conducted among 91 patients with chronic liver diseases, there was a significant increase intrahepatic expression of IL-17 in chronic HBV-related conditions than in HCs; furthermore, intrahepatic IL-17 was mainly localized in the fibrosis region, and its level correlated strongly with the degree of fibrosis (60). Other studies have also found increased intrahepatic IL-17+ cells or IL-17 levels, which correlate positively with fibrotic staging scores and clinical progression from CHB to cirrhosis, and most IL-17+ cells located in the fibrotic area (28, 44). In addition, intrahepatic IL-17 accompanied the higher serum IL-17 protein and mRNA levels in PBMCs, being higher among patients with cirrhosis than those with CHB, or HBsAg carriers (*P* < 0.01, for both) (44). Among HCV patients, a study evaluating the effect of HCV recurrence after orthotopic liver transplantation (OLT) showed that recipients with significant HCV-induced allograft fibrosis/cirrhosis and inflammation, presented higher frequency of HCV-specific Th17 cells, as well as proinflammatory mediators (IL-17, IL-1β, IL-6, IL-8, and MCP-1) (57).

In genetic studies, it has been noted that certain polymorphisms in IL-17 genes are more often present among those who progress to LC than in CHB patients (9, 14, 53); and are associated with a significant increase in LC risk either among monozygotic patients [OR 4.1, 95% CI: 1.4–11.84 or single allele carriers (OR 1.8, 95% CI: 1.16–2.9)] (14).

### The Th17/IL-17 Axis and the Response to Treatment in CVH

In CVH, the Th17/IL-17 axis status is associated with response to available treatments (23), as found in a study with 30 CHB patients, in which treatment with entecavir was associated with a significant decrease in the Th17 and Treg cell frequencies and related cytokines, in parallel to the reduction of HBV DNA load (61, 62). In other studies in CHB, the treatment either with telbivudine or with interferon-α resulted in the normalization of serum ALT and reduction or suppression of viral replication, associated with a significant decline in circulating Th17 cells and IL-17 levels (23, 54). Studies in HCV also found the same effect, as shown in a study including 27 HCV-infected patients, in which combined treatment with pegylated IFN-α and ribavirin resulted in a significant decrease in factors related to Th17 (IL-6 and IL-17), Th1 (IFN-α and MIP-1) responses, and profibrotic factors (FGF-b, VEGF); and this impact was principally in responder patients (62). In another study, Th17-related gene polymorphisms were associated with sustained responses to PEG-IFNa-2α (55).

In line with these findings, in an interventional study involving 56 cirrhotic patients allocated to autologous bone marrow mesenchymal stem cells (ABMSCs) transplantation or control group; transplantation significantly improved the liver function, accompanied by a marked decrease in Th17 cells and serum levels of proinflammatory cytokines (IL-17, TNF-α, and IL-6) (63).

### The Th17/IL-17 Axis and Mortality Prediction in CVH

Beyond its effect in predicting disease severity, the Th17/IL-17 axis elements are also predictors of mortality (56). In a study including 60 HBV-infected patients (30 with CHB and 30 with ACLF), the disproportionate increase of Th17 (compared to Treg) was associated with low survival among those with ACLF (64). These results have also been found in another study including 98 patients with HBV-related conditions (70 with CHB and 28 with LC), where the low Treg/Th17 ratio was associated with low survival among patients with cirrhosis and was associated with worse Child–Pugh and MELD scores (65). In another study, including 80 HBV-infected patients (40 with ACLF and 40 with CHB), the frequency of Th17 cells in peripheral blood, as well as IL-17 mRNA level in PBMCs, was significantly higher among ACLF patients who died than among survivors; and correlated positively with serum total bilirubin (*r* = 0.392, *P* = 0.012) and MELD score (*r* = 0.383, *P* = 0.015) (56). These findings suggest a significant contribution of this immune imbalance in disease severity and mortality.

### The Role of Other IL-17 Sources and Related Imbalances in CVH Outcomes

Besides the Th17 cells, recent investigations have found other immune cells, such as mast cells, and neutrophils as important sources of IL-17 in CVH, especially in late fibrosis stages (66–68). These findings emerge in a context after many studies have shown the domain of neutrophils as a predictor of outcomes in viral hepatitis and related diseases (19, 41). Therefore, these research together bring to light the importance of the interleukin-17 in pathogenesis and progression of CVH and can be one of the ways by which neutrophils, and other immune cell populations, exercise their pathogenic and predictive effect in CVH. Other Th17-related cytokines, such as IL-6 and IL-23, are involved in the evolution of CVH, regardless of stimulation of Th17 (11, 37, 69). The IL-23 and its receptor, in particular, have been found higher among HCV-infected patients than in control (mean 24.6 vs. 20.2 pg/mL; *P* = 0.005), with a positive correlation with ALT in HCV patients (69). Concerning the IL-6, its role was described in previous sections.

Besides increased Th17 cells, CVH has been associated with decreased or disproportionate count of regulatory T lymphocytes (Treg), another T helper subset, which is the functional counterbalance of Th17 cells (70); configuring a Treg/Th17 imbalance (13, 42, 64, 71). The balance between these two T helper lymphocytes subpopulations is fundamental (21, 22), and influenced by various factors (72–74). There is a plasticity and reciprocity between the two cell subpopulations that depends on environmental factors (70, 75, 76), being the proinflammatory environment, like that of the CVH, favorable to the polarization to Th17 cells (77, 78). The Treg/Th17 imbalance has been associated with greater hepatocellular damage in CVHs (21, 71, 79) and is related to advanced stages of cirrhosis and HCC (22, 65, 79), correlating inversely with severity scores and mortality (22, 64, 65). The Treg/Th17 imbalance has also been observed in other fibrosing liver diseases, such as autoimmune liver diseases (ALD) (80), biliary atresia (81), primary biliary cirrhosis (PBC) (82–84), non-alcoholic steatohepatitis (85, 86), schistosoma-induced hepatitis (87–89), and drug-induced hepatitis (90, 91).

Table 1 summarizes the clinical studies that evaluated the role of the Th17/IL-17 axis and related imbalances as drivers and predictors of outcomes in CVH.

**Table 1:** Clinical studies on the role of the Th17/IL-17 axis and associated imbalances in predicting outcomes in chronic viral hepatitis (CVH) and cirrhosis.

Reference	Virus	Biomarker	Patients	Results
Ge et al. (58)	Hepatitis B virus (HBV)	Subtype 17 of T helper lymphocytes (Th17)	30 patients with CHB and 20 matched controls	The percentage of Th17 cells in peripheral blood of CHB patients was significantly increased than in HCs (1.53 vs. 0.92%; *P* < 0.05); and kept a positive correlation with serum ALT in CHB patients

Ye et al. (71)	HBV	Th17, Treg, interleukin-4 (IL-4), and IFN-γ	88 liver specimens from HBV-infected patients, and six samples from controls	There was an increased intrahepatic frequency of IL-17-producing cells than IFN-γ-positive, IL-4-producing, and Treg cells. These cellular imbalances were higher in patients with severe hepatocellular damage than in mild

Wang et al. (92)	HBV	IL-23/IL-17	110 HBV-infected patients (39 with ACLF and 79 with CHB) and 32 HC	The IL-23/IL-17 pathway-related proinflammatory cytokines were found significantly increased in liver tissues of patients with HBV than HC

Niu et al. (22)	HBV	IL-17+/Treg ratio	57 patients with HBV-related liver failure (26 with CLF and 31 with ACLF) and 12 controls	The frequency of liver IL-17+ T cells was significantly higher in both HBV-related liver failure (CLF and ACLF) than in HC (*P* = 0.0001). The IL-17+/Treg ratio was significantly higher in ACLF and CLF than in HC (7.00, 4.33, and 0.00, respectively). The IL17+ T cells frequency correlated positively with total bilirubin (*r* = 0.579, *P* = 0.001) and MELD score (*r* = 0.367, *P* = 0.043)

Wang et al. (93)	HBV	IL-23, Th17, and IL-17	166 HBV-infected patients (108 with CHB and 58 with ACLF) and 62 controls, who underwent liver biopsies	There was increased intrahepatic expression of both IL-23 and IL-23R in HBV-infected livers than in controls, and the primary sources of IL-23 were liver myeloid dendritic cells and macrophages. In the presence of HBsAg or HBcAg, IL-23 efficiently stimulated the differentiation of naïve T CD4+ into Th17, that shown to be the primary source of IL-17

Zhai et al. (64)	HBV	Th17 and Treg cells	60 HBV-infected patients (30 with CHB and 30 with ACLF) and 30 controls	There was an increase of both Th17 and Treg cells in peripheral blood of ACLF patients. However, IL-17A was not regulated by Treg and the last exhibited significant inhibition of IFN-γ production. Most importantly, the low Treg/Th17 ratio was associated with low survival among ACLF patients

Chang et al. (42)	HCV	Th17	50 subjects with CHC and 23 HC	Compared to healthy individuals, patients had higher proportions of Th17 cells either circulating (1.56 vs. 0.96%, *P* < 0.001) or infiltrating the liver (16.08 vs. 0.82/hpf, *P* < 0.001); associated with higher serum IL-17 levels (84.86 vs. 60.52 pg/mL, *P* < 0.001). Both (circulating and intrahepatic) Th17 cells correlated with the severity of liver inflammation and damage

Wang et al. (56)	HBV	Th17 and IL-17 mRNA	80 HBV-infected patients (40 with ACLF and 40 with CHB) and 20 HC	The frequency of Th17 cells in peripheral blood, as well as IL-17 mRNA level in PBMCs, was higher in ACLF patients than in CHB (*P* = 0.045) or HC (*P* < 0.001). In addition, Th17 cells and IL-17 mRNA level were significantly higher among ACLF patients who died than in survivors. The frequency of Th17 cells correlated positively with serum TB (*r* = 0.392, *P* = 0.012) and MELD score (*r* = 0.383, *P* = 0.015) among ACLF patients

Foster et al. (49)	HCV	IL-17 and IL-22	157 HCV-infected patients (12 with acute infection, 134 with HCV-related fibrosis, and 11 with ESLD) and 41 HC	Chronic hepatitis C patients demonstrated an expansion of IL-17 and/or IL-22-producing T cell in hepatic compartment than in peripheral blood. Acute hepatitis C was not associated with a significant difference in IL-17 and/or IL-22-producing T cells expansion

Wu et al. (18)	HBV	Th17	133 subjects, HBV-infected (40 mild CHB, 37 severe CHB, and 20 AHB) and 36 HC	Patients with AHB and severe CHB had a higher frequency of Th17 cells in peripheral blood than those with mild CHB or HC (both *P* < 0.05). The increased peripheral Th17 correlated positively with ALT levels among severe CHB patients (*r* = 0.457, *P* = 0.004)

Wang et al. (56)	HBV	IL-17	91 patients with chronic liver conditions (55 with CHB, 42 with cirrhosis, 34 with HCC, 30 AsC) and 20 matched controls	There was a significantly increased intrahepatic expression of IL-17 in HBV-related chronic liver diseases. The intrahepatic IL-17 level correlated strongly with the degree of fibrosis, and it was mainly localized in the fibrosis region

Du et al. (44)	HBV	IL-17 and IL-17 mRNA	173 patients with CHB-related conditions (47 with CHB, 49 with cirrhosis, 44 with HCC, and 33 with CLF^a^)^b^ and 20 matched controls	Serum IL-17 protein and mRNA levels were significantly higher in the four CHB-related conditions than in controls (*P* < 0.001). Patients with cirrhosis exhibited the highest IL-17 concentrations in the serum and IL-17 mRNA in PBMCs. In addition, the levels of IL-17 in the liver tissues was higher in patients with cirrhosis than in those with CHB; and higher in this last than in HBsAg carriers (*P* < 0.01, for both)

Hao et al. (54)	HBV	Th17, IL-17, IL-22, and IL-23	24 CHB patients, who underwent treatment with telbivudine	Antiviral therapy was associated with a significant decline in circulating Th17 cells and IL-22 production, in parallel to the reduction of HBV DNA and normalization of serum ALT

Ashrafi Hafez et al. (69)	HCV	IL-23, and IL-27	64 patients with CHC and 37 matched controls	Serum level of IL-23 was higher in HCV-infected patients than in control group (mean 24.6 vs. 20.2 pg/mL; *P* = 0.005). There was a positive correlation between ALT and IL-23 in HCV-infected patients

Sun et al. (28)	HBV	Th17	78 patients with LC (Child A: 34; Child B: 22; Child C: 22), 23 with CHB, and 32 HC	Patients with cirrhosis had a significant increase in peripheral Th17 cells as increased disease severity (mean: 3.51, 3.94, and 4.46; for Child–Pugh A, B, and C, respectively). The plasma IL-17 concentration was significantly higher in LC patients than in HC (89.76 vs. 61.40; *P* < 0.01); and also, increased with disease severity. There was an increase in intrahepatic IL-17+ cells, which correlated positively with fibrotic staging scores and clinical progression from CHB to cirrhosis; and most IL-17+ cells were located in the fibrotic areas in the liver

Yang et al. (59)	HBV	Th17 and IL-17	96 patients with HBV-related conditions (20 AsC, 32 with CHB, 44 with ACLF), and 20 matched controls	Serum IL-17 concentration, intrahepatic, and peripheral Th17 cells were significantly higher in CHB and ACLF patients than in AsC and HCs; and increased as aggravated the immune inflammation from AsC, CHB, to ACLF. In addition, in ACLF patients, peripheral Th17 cells correlated positively with INR and MELD score

Yan et al. (94)	HBV	Th17 and Th1	150 patients with HCC (100 with HBV-related) who underwent blood and tissue samples	The levels of Th17 and Th1 cells were significantly higher in tumors of patients with HCC (*P* < 0.001) compared to corresponding non-tumor regions. The intratumoral density of IL-17-producing cells correlated inversely with OS (*r* = −0.784, *P* = 0.001); and predicted shorter DFS (median, 7.5 vs. 24.9 months, *P* = 0.03)

Yang et al. (95)	HBV	Th17, IL-17, and Treg	87 patients with HBV-associated conditions (40 with CHB, 27 with cirrhosis, and 20 with liver failure) and 20 HCs	The frequencies of Th17 in the peripheral blood were significantly higher in the patients with CHB, cirrhosis, and liver failure, compared with HC. The same trend was observed in the serum levels of IL-17. Both peripheral Th17 cells and serum IL-17 correlated positively with ALT and the prothrombin times

Feng et al. (96)	HBV	Th17 and Treg	96 HBV-infected patients, and 33 HC	Compared with controls, patients had higher Treg (6.80 vs. 4.42) and Th17 (6.15 vs. 2.66) in circulation. However, the Treg/Th17 ratio was significantly lower in patients (1.48 vs. 2.29, *P* = 0.0001); which suggests an ineffective suppression of proinflammatory response

Feng et al. (23)	HBV	Th17, IL-17, and Treg	22 CHB patients, who underwent treatment with interferon-α, and 30 HC	Compared with controls, patients had higher Th17 (3.94 vs. 2.66, *P* = 0.0436), IL-17 levels (16.88 vs. 8.59, *P* = 0.004), and Treg (5.72 vs. 4.42, *P* = 0.0019) at baseline. The suppression of viral replication induced by interferon-α was associated with a decrease in both Th17 cells, IL-17 levels, and Treg

Maggio et al. (25)	HCV	Peripheral Treg/Th17 balance	30 patients with CHC and 30 with NAFLD/NASH who underwent NLCD for 30 days	After 30 days of NLCD, CHC patients showed a significant reduction in Th17 cells frequency, and an increase in the percentage of Treg cells, thus improving Treg/Th17 balance. The decrease in Th17 cells correlated with a decline in IL-17 and IL-22 serum levels

Wang et al. (9)	HBV	IL-17A, and IL-17F gene polymorphisms	433 subjects (130 with CHB, 132 with HBV-related cirrhosis, and 71 controls)	There was a significant increase in the risk of cirrhosis among subjects carrying the IL-17A rs4711998 G allele (OR = 1.54, *P* = 0.025) and those with the IL-17A rs4711998 AG genotype (OR = 1.75, *P* = 0.02) compared with other polymorphisms

Ge et al. (14)	HBV	IL-17A and IL-17F gene polymorphisms	331 patients with HBV-related conditions (163 LC and 168 CHB) who underwent gene polymorphisms analysis	The frequency of IL-17A G197A genotype AA was significantly higher in LC that in CHB patients (42.33 vs. 27.98%, *P* = 0.032), as well as the allele A (56.34 vs. 46.15%, *P* = 0.011). There was a significant increase in LC risk either among AA genotype patients (OR 4.186, 95% CI: 1.479–11.844) or one allele carriers (OR 1.856, 95% CI: 1.161–2.967)

Tian et al. (61)	HBV	Th17, Treg, Th1, Th2, and related cytokines	30 CHB patients, who underwent treatment with Entecavir	Antiviral therapy was associated with a significant decrease in the Th17 and Treg cell frequencies and related cytokines, in parallel to the reduction of HBV DNA load. By contrast, the treatment was associated increase in the Th2 cell and related cytokines

Zhao et al. (97)	HBV	TLR2, Th17, and IL-17	34 patients with HBV infection (10 acute and 24 CHB) and 15 matched controls	The proportion of Th17 cells among PBMCs was significantly higher in CHB (1.78%) than either AHB patients (1.28%, *P* = 0.0004) or controls (0.78%, *P* = 0.0009). After stimulation with HBV envelope peptides, the expression of TLR2 and IL-17A in T lymphocytes was remarkably increased in CHB patients than in AHB. In addition, the stimulation of PBMCs with a TLR2 agonist induced a higher frequency of Th17 cells in CHB patients than AHB patients (1.39 vs. 0.68%, *P* = 0.002); with the increased IL-17 production

Xue-Song et al. (79)	HBV	Th17, Treg, and IL-17	48 patients with chronic HBV (12 AsC, 18 CHB, and 18 ACLF), 10 with AHB, and 10 HC	Compared to HC, both AHB and ACLF patients favored the Th17 cells differentiation, accompanied by a higher proportion of peripheral Th17 cells and high level of serum IL-17A (*P* < 0.01 for both). Both Th17 frequency and plasma IL-17A levels correlated positively with ALT and TB levels among those with CHB. In asymptomatic HBV carriers, by contrast, there was a favoritism to Treg differentiation. Both CHB and ACLF had lower Treg/Th17 ratio than in HC (*P* < 0.05); and this correlated inversely with TB levels (*r* = −0.41, *P* = 0.004)

Yu et al. (65)	HBV	Treg/Th17 ratio and TGF-β1/IL-17 ratio	98 patients with HBV-related conditions (70 with CHB, 28 with LC) and 20 controls	Patients with LC, especially non-survival ones, presented a significant decrease in the Treg/Th17 ratio. The lower Treg/Th17 ratio was associated with worse Child–Pugh and MELD scores, which suggests that the dominance of Th17 over Treg has a significant contribution in disease severity

Shi et al. (98)	HBV	IL-17 and IL-17 mRNA	123 patients with HBV-related conditions (30 with CHB, 79 with LC, 14 with severe CHB), and 20 controls	IL-17 mRNA expression levels in PBMCs were significantly higher in patients with HBV-conditions than in the controls. PBMCs IL-17 mRNA and the serum IL-17 protein were significantly higher in patients in higher Child–Pugh (B or C) than in lower scores. Serum IL-17 levels correlated positively with TB, ALT, and Child score; and correlated inversely with albumin

Wang et al. (99)	HBV and HCV	Treg/Th17	38 patients with ESLD (33 due to viral cause) who underwent liver transplantation	The frequency of circulating Th17 cells was significantly increased in liver allograft recipients who developed acute rejection; whereas Tregs, and consequently the Tregs/Th17 ratio, was significantly decreased in these patients. The level of Th17 cells had a positive correlation with RAI (*r* = 0.890, *P* < 0.001).

Xu et al. (63)	HBV	Treg/Th17 balance	56 patients with LC who were randomly assigned to ABMSCs transplantation or control group	After 24-week follow-up, 20 cases and 19 controls completed the study. There was a significant increase in Treg and a marked decrease in Th17 cells in the transplantation group compared with control, leading to an increased Treg/Th17 ratio. In addition, ABMSCs transplantation improved patients’ liver function and significantly decreased the serum levels of proinflammatory cytokines (IL-17, TNF-α, and IL-6)

Jimenez-Sousa et al. (62)	HCV	Th17 and Th1 factors	27 HCV-infected patients who underwent 12 weeks’ treatment with pegylated IFN-α and ribavirin and 10 HC	HCV infection induced the secretion of chemokines and cytokines involved in both Th1 (like IFN-α and MIP-1) and Th17 responses (such as IL-6 and IL-17), and two profibrotic factors (FGF-b, VEGF). Compared to the control group, these increases reached significances as follow: for MIP-1 alpha (4.7-fold), TNF-α (3.0-fold), FGF-b (3.4-fold), VEGF (3.5-fold), and IL-7 (5.6-fold). The 12 weeks combined treatment resulted in a significant down-modulation of the secretion of key Th1 and Th17 proinflammatory or profibrotic factors principally in responder patients

Zhang et al. (20)	HBV	Th17	83 HBV-infected patients (66 with CHB, 23 with ACLF) and 30 HC	Patients presented higher frequency of circulating Th17 than HC; and increased with disease progression (mean, 2.42, 4.34, and 5.62% for HC, CHB, and ACLF, respectively). Both circulating and intrahepatic Th17 cells correlated positively with serum ALT, and histological activity index

Basha et al. (57)	HCV	Th17, Treg, IL-17	60 OLT recipients (51 HCV+, 9 HCV−) and, 15 HC	Recipients with recurrent HCV-induced allograft inflammation and fibrosis/cirrhosis presented a significant increase in the frequency of HCV-specific CD4+ Th17 cells; as well as proinflammatory mediators (IL-17, IL-1β, IL-6, IL-8, and MCP-1). Recurrent patients despite demonstrating increased Treg frequency, this did not inhibit HCV-specific CD4+ Th17 cells

Hu et al. (100)	HBV	IL-21 and IL-21-secreting CD4+ T cells	79 patients with CHB (39 with ACLF, 20 with moderate, and 20 with severe disease) and 10 HC	The frequencies of IL-21-secreting CD4+ T cells were higher in ACLF (4.84%) and severe CHB (4.18%) than in moderate CHB patients (1.38%) or HC (1.10%) (*P* < 0.002 for all). Serum IL-21 levels were highest in ACLF group compared with severe or moderate CHB patients, or HC (median 96.7, 65.2, 62.3, and 50.1 pg/mL, respectively; *P* < 0.001 for all). Serum IL-21 was associated with high MELD score (*P* = 0.001) and mortality (*P* = 0.038); and recovery from ACLF was associated with a reduction in its levels (*P* = 0.003). The stimulation with rhIL-21 significantly increased the secretion of other proinflammatory cytokines (IL-1β, IL-6, and TNF-α) by PBMCs

*ABMSCs, autologous bone marrow mesenchymal stem cells; ACLF, acute-on-chronic liver failure; AHB, acute hepatitis B; ALT, alanine aminotransferase; AR, acute rejection; AsC: asymptomatic surface antigen carriers; CHB, chronic hepatitis B; CLF, chronic liver failure; DFS, disease-free survival; ESLD, end-stage liver disease; FGF-b, fibroblast growth factor-basic; HC, healthy controls; Hpf, high-powered fields; INR, international normalized ratio; MIP-1, macrophage inflammatory protein-1; NLCD, normocaloric low cholesterol diet; OLT, orthotopic liver transplantation; OS, overall survival; PBMCs, peripheral blood mononuclear cells; RAI, rejection activity index; rhIL-21, recombinant human interleukin-21; TLR2, toll-like receptor 2; TB, total bilirubin; VEGF, vascular endothelial growth factor; WBC, white blood cell*.

*^a^The study included liver biopsy samples obtained from CHB (*n* = 57) and cirrhosis (*n* = 31) patients*.

*^b^Control liver biopsy samples were obtained from asymptomatic HBsAg carriers (AsC, *n* = 35)*.

## Mechanisms and Pathways Linking the Th17/IL-17 Axis to Fibrogenesis and Cirrhosis in CVH

### The Th17 Differentiation and Th17-Secreted Cytokines

Overall, Th17 cells differentiate from naive T helper cells, in response to a variety of stimuli, in the presence of key cytokines, namely IL-1β, IL-6, IL-21, IL-23, and TGF-β (101–105). In viral hepatitis, the virus particles are recognized by toll-like receptor (TLR2 and TLR4) present on the surface of the antigen-presenting cells (dendritic cells, macrophages, and monocytes) that result in their activation (97, 106). These activated cells, using the nuclear factor kappa B (NF-κB) and/or mitogen-activated protein kinase (MAPK) signaling pathways, produce the proinflammatory cytokines IL-1, IL-6, IL-21, and IL-23 (38, 93, 107) that drives the Th17 differentiation (93, 104, 107, 108). In the particular case of HCV, there are two additional pathways: the first one consists in the production of the thymic stromal lymphopoietin (TSLP) by HCV-infected hepatocytes, in an NF-κB-dependent process, and is this hepatocyte-derived TSLP that enhances activated APCs to produce the IL-1, IL-6, IL-21, and IL-23 (109). The second consists in particular evidence that HCV core protein exerts a function of a toll-like receptor 2 ligand, promoting, by itself, the activation of the APCs, the production of inflammatory cytokines that favor Th17 differentiation, and the evasion of the immune system (4, 12, 27). After being differentiated, the Th17 cells secrete its cytokines (IL-17, IL-21, and IL-22), being the IL-17 the main driver of a chain of events that have in common the favoritism of the proinflammatory and profibrotic pathways (13, 47, 49).

### The Role of IL-17 Axis, and Associated Signaling Pathways, in Liver Fibrogenesis and Cirrhosis

The subtype 17 of T helper lymphocyte cells is increased in almost all chronic and fibrosing liver diseases, including ALD, such as autoimmune hepatitis (AIH) (50, 110, 111), primary sclerosing cholangitis (PSC) (112, 113), PBC (16, 83, 114, 115); biliary atresia (29, 81, 116), non-alcoholic steatohepatitis (85, 117, 118), and viral hepatitis (42, 44, 58, 109). These findings reveal the pivotal role of the Th17/IL-17 axis in liver fibrogenesis. However, the stimuli that attract Th17 cells to the liver are not completely elucidated. What is known is that injured liver cells secrete a variety of chemokines, such as CXCL9, CXCL10, and CCL20 (119, 120), that drive the recruitment of Th17 cells to the liver, binding to their receptors (CXCR3 and CCR6) expressed in Th17 cells (16, 119–123). This aggregate of chemokines and their receptors seems to determine, the differential cellular recruitment (32, 123, 124), the disposition of the Th17 cells within fibrosis areas (16, 60, 119, 120); and CXCL10, in particular, has itself a profibrotic effect influencing in the number and activity of HSCs, and participating in the cross talk between hepatocytes, HSCs, and immune cells (124–126).

In the liver, Th17 cells produce their cytokines (93), with IL-17 being the most associated with the progression of cirrhosis (13, 28, 49). There are receptors for IL-17 expressed in hepatocytes, in the liver sinusoids endothelial cells, in hepatic stellate cells (HSCs), and Kupffer cells (KC) (13). The functional implication of IL-17 in liver tissue is well characterized in the activation and/or stimulation of HSCs and KC (13, 17, 127) and cholangiocytes (115, 116).

#### Hepatic Stellate Cells

As already said, stellate cells express receptors for IL-17 (IL-17RA and IL-17RC) on their surface (13). The stimulation with IL-17 induces the rapid translocation of transcription factors NF-κB and Stat3 to the cellular nucleus (13), where activate the gene transcription of proinflammatory cytokines (IL-6 and TNF-α) and profibrotic factors (TGF-β1) (17, 50). In addition, IL-17 promotes the proliferation of HSCs, the upregulation of TGF-β receptor, IL-17RA, and IL-17RC (13, 16, 127). So, the IL-17 has the property to induce the activation of HSCs and fibrogenesis, and this effect seems to be synergistic with that of IL-6 and TNF-α (13, 128).

In the liver tissue, particularly in HSCs, the IL-17 also presents the following effects: increases the genic expression of type I collagen and induces its production through TGF-β, or Stat3/SMAD2/3 signaling pathways (13, 127). In addition, IL-17 upregulates matrix metalloproteinases (MMP2, MMP3, and MMP9) expression *via* NF-κB and Stat3 signaling pathways (13, 127, 129) and increases the expression of tissue inhibitor of matrix metalloproteinase I (TIMP1) and the production of related proteins (127). The combination of these effects results in increased production of extracellular matrix and changes in its degradation. This role of IL-17 has been reinforced in experimental model where liver fibrosis was inhibited or attenuated in IL-17RA−/− mice exposed to carbon tetrachloride (CCl4) or subjected to bile duct ligation (BDL), associated with reduced mRNA expression of fibrogenic genes (collagen-α1, MMP3, TIMP1, and TGF-β1) (13, 130). The role of IL-17 on MMPs and related signaling pathway has also been found in other organs such as the heart (131).

#### IL-17, TGF-β, and Induction of Cellular Transition/Transdifferentiation

In addition to the direct effects, described above, the IL-17 cooperates with TGF-β1 to induce the activation of HSCs and their transition into a proliferative, contractile, and fibrogenic phenotype—the myofibroblast (13, 132, 133). These events also lead to an excessive synthesis of ECM and the contractility of myofibroblasts resulting in changes in the hepatic microarchitecture and microcirculation (134–136). The IL-17 also has the effect of inducing epithelial–mesenchymal transition (EMT) in hepatic tissue as observed in hepatocytes of patients with HCC (137) and biliary epithelial cells as seen in PBC (115). It is important to emphasize that the exposure of the hepatic tissue to IL-17 increased the expression of TGF-β in almost all liver resident cells (13, 127). Moreover, the TGF-β is well documented that induces the EMT of the hepatocytes (138–140). Therefore, we can deduce that the IL-17, through the induction of TGF-β production, is an indirect promoter of EMT in the liver (13, 132, 137, 139). This effect of IL-17 in EMT is well described in many other organs (and clinical conditions), as in the respiratory epithelium (141, 142) and prostate (143); and has been proven to be a process dependent on TGF-β or NF-κB pathways (115, 141, 142, 144).

The activation of HSCs is associated with two other Th17/IL-17 axis-related changes. The first results from the rarefaction of retinoic acid in HSCs. Under normal conditions, the quiescent HSCs have many granules containing retinoic acid (135), which acts inhibiting the Th17 and favoring the Treg cells differentiation, with a protective effect on the liver (145, 146). However, when activated HSCs undergo changes in their metabolism and retinoic acid content, which affects the differentiation of Treg cells and, consequently, the loss of the protective effect (135, 147, 148). The second results from the evidence that activated HSCs exacerbate liver fibrosis by enhancing IL-17A production by T cells, in a TLR3-dependent manner (45), that in combination with the rarefaction of retinoic acid results promoting further Treg/Th17 imbalance and fibrogenesis (45, 149).

#### Kupffer Cells

In KC, as well as in HSCs, stimulation with IL-17 led to the production of proinflammatory cytokines and TGF-β1, through NF-κB and Stat3 pathways (13, 150–153). It also upregulates the receptors of TGF-β, IL-17A, and IL-17C and promotes the further production of IL-17A, IL-17F (13, 127). In addition, under stress conditions, KC are involved in increased differentiation of Th17 and decreased Treg, through an IL-6-dependent mechanism, promoting further Treg/Th17 imbalance and perpetuating the proinflammatory and fibrogenic consequences of this axis (43, 154).

### The Role of IL-17 in the Systemic Circulation and Its Repercussions in the Liver Tissue

In the systemic circulation, the IL-17 sustains the proinflammatory environment, stimulating the granulopoiesis through the release of granulocyte-macrophage colony-stimulating factor (GM-CSF) (33, 155–158). On inflammatory cells (either in systemic circulation or infiltrating the liver), the IL-17 induces the release of the IL-6 (159). The elevation of the IL-6 contributes to further Th17 responses because it promotes its differentiation (104), as IL-6 receptors are expressed on the surface of CD4+ T cells (43, 51). This process also occurs using the STAT3, NF-κB, and MAPK signaling (43, 151).

**Figure 2:**
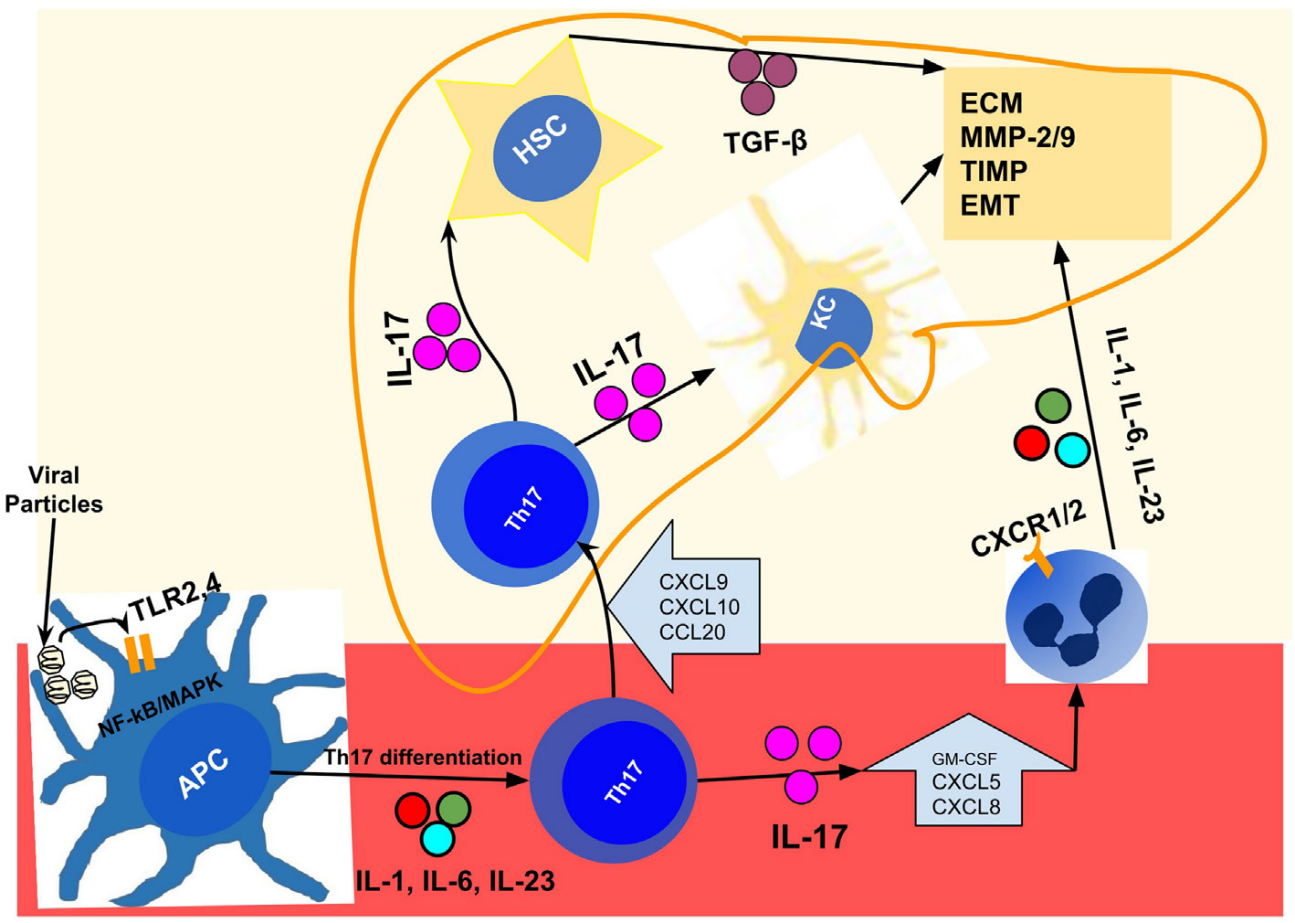
Representation of events chain from viral injury to Th17/IL-17 axis activation, liver fibrogenesis, and cirrhosis in chronic viral hepatitis. After infection by hepatitis B virus/hepatitis C virus; distinct viral particles are recognized byTLR2 and TLR4 present on the surface of the APCs (dendritic cells, macrophages, and monocytes), which result in their activation (97, 106). These activated cells, using the NF-κB and MAPK signaling pathways, produce the proinflammatory cytokines IL-1, IL-6, IL-21, and IL-23 (38, 93, 107) that drive the Th17 differentiation and IL-17 production in peripheral blood (93, 104, 107, 108). In the liver, injured cells secrete a variety of chemokines like CXCL9, CXCL10, and CCL20 (119, 120) that drive the recruitment of Th17 cells to the liver, through binding to their receptors (CXCR3 and CCR6) expressed in Th17 cells (120–123). Intrahepatic Th17 cells and IL-17 are responsible for hepatic stellate cell activation (13, 17), increased expression of TGF-β (127), MMP (13, 127, 130), collagen synthesis (13, 127), and induction of EMT (115, 137, 142). In addition, it promotes the recruitment of other inflammatory cells (32, 160) through the release of chemokines such as CXCL5 and CXCL8/IL-8 (156, 160, 161) whose receptors (CXCR1 and CXCR2) are abundantly expressed in neutrophils, monocytes, and macrophages (32, 125, 161–164). APCs, antigen-presenting cells (dendritic cells, macrophages, and monocytes); EMT, epithelial–mesenchymal transition; HSC, hepatic stellate cell; IL-1, interleukin-1; IL-17, interleukin-17; IL-21, interleukin-21; IL-23, interleukin-23; IL-6, interleukin-6; KC, Kupffer cells; MAPK, mitogen-activated protein kinase; MMP, matrix metalloproteinases; NF-κB, nuclear factor-kappa B; TGF-β, transforming growth factor beta; Th17, T helper lymphocytes, subtype 17; TLR2, toll-like receptor 2; TLR4, toll-like receptor 4.

The repercussion at the organic level is the liver infiltration by inflammatory cells that occurs because IL-17 promotes endothelial activation (32, 33, 47), and release of attracting chemokines such as CXCL5 and CXCL8/IL-8 (156, 160, 161) whose receptors (CXCR1 and CXCR2) are abundantly expressed neutrophils and monocytes (32, 125, 161–164). Once in the liver, neutrophils are involved in various points of the fibrogenesis chain, including the release MMP9 (165–167) and seem to affect the functioning of the TIMP-1 (168). Recent studies have shown that neutrophils released itself the IL-17, mainly in the advanced stages of fibrosis (66); and there is a sustained cross talk between neutrophils and Th17 cells (32, 164). Figure 2 represents the chain of events from viral injury, Th17/IL-17 axis activation, to liver fibrogenesis and cirrhosis.

### The Role of IL-17 Axis in Other Liver Diseases and Other Organs Fibrosis

The role of Th17/IL-17 axis in hepatic fibrosis has been found in various liver diseases such as NASH (117, 118), obstructive cholestasis (169), PSC (112), PBC (16, 114, 115), biliary atresia (29, 116), drug-induced (91, 170), protozoa-associated cirrhosis (87, 171, 172), and viral hepatitis (20, 30, 44, 173). Moreover, the role of Th17/IL-17 axis extends to related diseases such as HCC (94, 174, 175), which suggests a continuous, or at least related parts of a whole, in the pathogenesis of these conditions (53, 94, 174, 175). Additional evidence comes from observational studies that have found an increased occurrence of hepatic inflammation associated with dysregulated protein/lipid metabolism, and progression to cirrhosis in clinical conditions such as psoriasis that have the Th17/IL-17 axis hyperactivation as the crucial point in their pathogenesis (48, 176).

It is worth noting that HCV infection induces the development of autoantibodies to liver autoantigens, in up to 10% of patients, in addition to previously described mechanisms, leading to an overlap between CVH and AIH (177–180). Certainly, the Th17/IL-17 axis plays a pivotal role in the progression to cirrhosis regardless of the dominant mechanism in the pathogenesis (20, 49, 111, 181, 182).

The role of the Th17/IL-17 axis has been found in other diseases with a fibrosing behavior in various organs such as intestine (31, 183), lung (184, 185), and peritoneum or kidney (46, 186); and its block has shown to prevent/mitigate the fibrosis in many pathological conditions (187–189).

### Hepatoprotective Agents Targeting the Th17/IL-17 Axis, Associated Cytokines, and Signaling Pathways

As we have seen the Th7/IL-17 axis is involved in several known points of fibrogenesis chain, including the activation of HSCs (17), recruitment of inflammatory cells (32, 160), expression of proinflammatory and profibrotic cytokines (127), in stimulus to collagen synthesis, and in the imbalance between MMP and TIMPs (13, 127, 133). Added to this is the parallel evidence of being a promoter of EMT or myofibroblast transition (115, 137, 142). Therefore, the inhibition of Th17/IL-17 axis (and its signaling pathways) represents a promising strategy in the treatment of organic fibrosis.

#### Agents That Downregulate the Th17/IL-17 Axis or Restore the Treg/Th17 Balance

There are a variety of agents targeting these immune pathways, with the potential to slow down the CVH-induced cirrhosis. From those that downregulates the Th17/IL-17 axis to those that restore the Treg/Th17 balance. Brief citations are made about some of these agents that have shown benefit in preliminary studies, such as cannabinoid receptor 2 agonists, whose action is to counteract immune and fibrogenic responses induced by interleukin-17 (190). Another treatment that ameliorates hepatic fibrosis by regulation of Treg/Th17 cells, and downregulating the IL-17, is bone marrow-derived stem cells transplantation (63, 191). It is also worth to cite the vitamin D and analogs that inhibit the development of liver fibrosis by reducing the Th17 differentiation and IL-17 production, and activate the Treg differentiation (186, 192–194). The rapamycin demonstrated to improve hepatic fibrosis that was associated with a decrease of Th17 and expansion of Treg (91, 195). In another study, the administration of an mTOR inhibitor decreased the IL-17 production induced by IL-6; and this effect occurred through decreasing mTOR/STAT3 activation (43). Halofuginone, an inhibitor of Th17 cells differentiation (196); has shown to be a promising antifibrotic drug, and its impact in reducing liver fibrosis severity is associated with a significant reduction in Th17 cells and related cytokines (197). Another compound with protective effects in liver fibrosis is the polyphenolic molecule mongol that inhibits Th17 cells differentiation and suppresses HSCs activation (198).

Still, about Treg/Th17 balance restoration, adoptive transfer of Tregs ameliorated the severity of liver injury, accompanied by increased levels of hepatic Treg and IL-10 as shown in a model of Triptolide-induced liver injury (90). On the other hand, some drugs with use and efficiency established in viral hepatitis, such as interferon, appear to have effects that involve this axis, among its various mechanisms (24, 62, 187).

#### Agents Targeting the Receptors and Signaling Pathways Involved in Th17/IL-17 Axis Effects

The inhibition of the activation of HSCs is, undoubtedly, one of the most attractive strategies to slow down fibrogenesis (199). Multiple receptors and signaling pathways are involved in the chain of events of Th17/IL-17 axis-mediated HSCs activation, and can also be therapeutic targets. So, agents such as resveratrol, curcumin, Dioscin, the flavonoid quercetin, and other agents have emerged as inhibitors of the HSCs activation targeting the TLR3, TLR2/4, STAT3, and/or MAPK/NF-κB, the main receptors and pathways in Th17/IL-17 axis-mediated HSCs activation (200–207). Agents such as rosiglitazone and rapamycin demonstrated the potential to interfere with the fibrogenic pathways by reducing the expression of TGF-β (195, 208). Others such as the ruxolitinib have also shown the potential to inhibit the hepatotoxicity and fibrogenesis, after initial injury by multiple agents, by inhibiting the JAK/STAT pathway (207, 209, 210).

## Conclusion and Future Directions

The imbalanced immunity at Th17/IL-17 axis level plays a significant role in liver fibrogenesis after initial HCV or HBV injury. The Th17/IL-17 axis drives of a chain of events that promote a proinflammatory and profibrotic environment by recruiting neutrophils and monocytes and by inducing the expression and production of interleukin-23 and IL-6 either in the liver or peripheral cells. All resident liver cells express receptors for IL-17; and liver cells respond to the IL-17 exposition by increasing the expression of profibrotic and proinflammatory factors such as TGF-β, MMPs, and TIMP. In addition, IL-17 induces the transformation of HSCs to myofibroblasts and the EMT of the hepatocytes, promoting the synthesis of extracellular matrix, cell contractility, and all changes in the liver microstructure and microcirculation.

## Author Contributions

FCP: prepared the manuscript text and figures.

## Conflict of Interest Statement

The author declares that the research was conducted in the absence of any commercial or financial relationships that could be construed as a potential conflict of interest.
